# Two potential hookworm DAF-16 target genes, SNR-3 and LPP-1: gene structure, expression profile, and implications of a *cis*-regulatory element in the regulation of gene expression

**DOI:** 10.1186/s13071-014-0609-0

**Published:** 2015-01-08

**Authors:** Xin Gao, Kevin Goggin, Camille Dowling, Jason Qian, John M Hawdon

**Affiliations:** Current affiliation: The Genome Institute at Washington University, 4444 Forest Park Ave, St. Louis, MO 63108 USA; Department of Microbiology and Tropical Medicine, The George Washington University Medical Center, Washington, DC USA

**Keywords:** Hookworm, Gene expression, DAF-16/FOXO, Regulatory elements

## Abstract

**Background:**

Hookworms infect nearly 700 million people, causing anemia and developmental stunting in heavy infections. Little is known about the genomic structure or gene regulation in hookworms, although recent publication of draft genome assemblies has allowed the first investigations of these topics to be undertaken. The transcription factor DAF-16 mediates multiple developmental pathways in the free living nematode *Caenorhabditis elegans*, and is involved in the recovery from the developmentally arrested L3 in hookworms. Identification of downstream targets of DAF-16 will provide a better understanding of the molecular mechanism of hookworm infection.

**Methods:**

Genomic Fragment 2.23 containing a DAF-16 binding element (DBE) was used to identify overlapping complementary expressed sequence tags (ESTs). These sequences were used to search a draft assembly of the *Ancylostoma caninum* genome, and identified two neighboring genes, *snr-3 and lpp-1*, in a tail-to-tail orientation. Expression patterns of both genes during parasitic development were determined by qRT-PCR. DAF-16 dependent *cis*-regulatory activity of fragment 2.23 was investigated using an *in vitro* reporter system.

**Results:**

The *snr-3* gene spans approximately 5.6 kb in the genome and contains 3 exons and 2 introns, and contains the DBE in its 3′ untranslated region. Downstream from *snr-3* in a tail-to-tail arrangement is the gene *lpp-1*. The *lpp-1* gene spans more than 6 kb and contains 10 exons and 9 introns. The *A. caninum* genome contains 2 apparent splice variants, but there are 7 splice variants in the *A. ceylanicum* genome. While the gene order is similar, the gene structures of the hookworm genes differ from their *C. elegans* orthologs. Both genes show peak expression in the late L4 stage. Using a cell culture based expression system, fragment 2.23 was found to have both DAF-16-dependent promoter and enhancer activity that required an intact DBE.

**Conclusions:**

Two putative DAF-16 targets were identified by genome wide screening for DAF-16 binding elements. *Aca*-*snr*-3 encodes a core small nuclear ribonucleoprotein, and *Aca-lpp-1* encodes a lipid phosphate phosphohydrolase. Expression of both genes peaked at the late L4 stage, suggesting a role in L4 development. The 3′-terminal genomic fragment of the *snr-3* gene displayed *Ac*-DAF-16-dependent *cis*-regulatory activity.

**Electronic supplementary material:**

The online version of this article (doi:10.1186/s13071-014-0609-0) contains supplementary material, which is available to authorized users.

## Background

Hookworm disease is a major public health problem in large portions of Asia, Africa and South America [1]. The disease is caused by parasitic hookworms that have a complex life cycle involving free-living stages and host-dwelling stages [2]. The transformation of the hookworm from one developmental stage to another is accompanied by many drastic changes in morphological, physiological, and biochemical properties [3,4]. Faithful execution of these developmental processes requires a precise and carefully orchestrated series of steps dependent on the proper activation/inactivation of a selected group of genes, the so-called parasitism genes [4,5].

Gene expression begins with transcription initiation. Multiple proteins, known as transcription factors, bind to specific DNA sequences. This binding event can activate or inhibit the transcription machinery so that gene expression is appropriately regulated [6]. Practically nothing is known about the regulation of gene expression in hookworms. However, this situation is expected to change with the rapidly increasing hookworm genomic information [7-9]. Specifically, the recent published *Necator* genome draft [9] and other nearly completed *Ancylostoma* hookworm genome projects (Mitreva, personal communication, 2013), serve as a good starting point for studying transcriptional regulatory elements, and provide opportunities to address how stage specific gene expression patterns are established in hookworms.

We have long been interested in understanding the critical genes and pathways relevant to the parasitism establishment process of hookworms. Our previous studies demonstrated that insulin-like signaling (ILS) was required in the transition to parasitism of infective third-stage hookworm larvae (L3) and identified the first hookworm molecule in this pathway, a forkhead transcription factor *Ac*-DAF-16, from *Ancylostoma caninum* [10-12]. We showed that *Ac*-DAF-16 was transcriptionally active through a consensus DAF-16 binding element (DBE) and capable of interacting with the molecular chaperone *Ac*-FTT-2 in a phosphorylation-site dependent manner [12]. We next reported the isolation of a small set of hookworm genomic fragments that bound to the *Ac*-DAF-16 DNA binding domain (DBD), and took advantage of the availability of the hookworm genome and transcriptome data to further analyze the digital expression profiles of the linked transcripts [13,14]. The predicted functions of those linked transcripts suggest that hookworm DAF-16, similar to its *C. elegans* and mammalian orthologs, is involved in diverse biological processes. *C. elegans* DAF-16/FOXO has been postulated to mediate such diverse biological roles by regulating expression of different subsets of genes in response to different stimuli [15-17].

To better decipher how hookworm DAF-16 mediates its diverse biological functions, a more comprehensive characterization of hookworm DAF-16 target genes is required. In our genome-wide screening of *Ac*-DAF-16 binding sites and the subsequent informatic searches for the aligned ESTs surrounding those binding sites, a hookworm ortholog of the *Caenorhabditis elegans* small nuclear ribonucleoprotein-3 (SNR-3/snRNP D1), *Ac*-SNR-3, was identified as a potential *Ac*-DAF-16 downstream target. *Ac-snr-3* gene is linked to an *Ac*- DAF-16 bound genomic fragment which is located at its 3′-end, and this genomic fragment (Fragment2.23) contained a DBE (DAF-16 family binding element) [13]. With the subsequent availability of improved *A. caninum* genome scaffold information, we were able to further analyze the genome sequence surrounding Fragment 2.23 and identified another linked gene, lipid phosphate phosphatase (LPP-1).

SNR-3 is one of seven conserved Sm proteins that form a heptameric core complex required for the biogenesis and function of the small nuclear ribonucleoproteins (snRNPs) involved in catalyzing mRNA splicing [18-20]. LPP-1 catalyzes the dephosphorylation of various lipid phosphate substrates and might function during stress conditions to regulate specific cellular pools of lipid-signaling molecules or cell signaling through lipid phosphates [21-23]. In *C. elegans*, both SNR-3 and LPP-1 have been implicated in controlling development, as evidenced by their association with extremely pleiotropic loss-of-function phenotypes affecting embryonic viability, larval viability, and fertility when gene specific RNAi was applied [24-27].

The relative locations of *Aca-snr-3* and *Aca*-*lpp-1* to the identified DBE and their importance in worm development have led us to investigate these two genes in more details. In the present study, we analyzed gene structures of hookworm *snr*-3 and *lpp-1* and their phylogenetic relationship to the counterpart proteins from other species. The expression profiles of hookworm SNR-3 and LPP-1 were also characterized across post infection developmental stages. The genome Fragment 2.23 was shown to increase the basal luciferase gene expression compared to the controls in the presence of *Ac*-DAF-16, and the DBE element in this fragment mediates this effect. This study represents the first post-infection exploration of gene expression in hookworms, and the first functional analysis of a regulatory element present in the hookworm genome.

## Methods

### Sequence analysis of hookworm *snr-3* and *lpp-1*

Hookworm expressed sequence tags (ESTs), cDNA sequences, genomic scaffolds and contigs were obtained from Nematode.net (http://www.nematode.net) or Genbank at National Center for Biotechnology Information (NCBI) (http://www.ncbi.nlm.nih.gov/genbank/). Data used to generate the gene models were from a draft *A. caninum* genome assembly (Newbler June 2010) found at Nematode.net (http://www.nematode.net/NN3_frontpage.cgi?navbar_selection=home&subnav_selection=Acan_newbler_assembly). Local DNA databases of hookworm genome scaffolds and ESTs were created and queried using BioEdit Sequence Alignment Editor Version 7.0.5.3 [28]. The open reading frame and untranslated regions of the cDNA were predicted based on homology searches using BLASTX and BLASTP programs at NCBI. Intron-exon boundaries were located by comparison of ESTs and genomic DNA sequences using Bioedit. Multiple alignments were performed and displayed using the Clustal W and BOXSHADE programs available on the ExPASy Bioinformatics Resource Portal (http://www.expasy.org/). The phylogenetic tree was inferred using the Maximum Likelihood method based on the JTT matrix-based model [29] in MEGA6 [30]. Transcription factor bind sites were predicted using JASPAR (http://jaspar.genereg.net/) [31] and the TFSEARCH server (http://www.cbrc.jp/research/db/TFSEARCH.html).

### Parasite culture and sorting

The Indian strain of *Ancylostoma ceylanicum* (US National Parasite Collection #102954.00) was maintained in beagles as previously described [32]. Animals were housed and treated in accordance with institutional animal care and use committee guidelines at The George Washington University (protocol A147). Infective *A. ceylanicum* L3s were recovered from coproculture by modified Baermann technique after incubation at 22°C for approximately one week, and stored for periods up to 5 weeks in BU buffer (50 mM Na_2_HPO_4_/22 mM KH_2_PO_4_/70 mM NaCl, pH6.8) [33], at room temperature until use for infection or cDNA preparation.

Four- to five-week-old Syrian golden hamsters were inoculated orally using a feeding needle with 2000 *A. ceylanicum* infective L3s for recovery of early parasitic stages, or 100 L3s for recovery of later stages (L4 and adult). Hookworms were collected from intestines of animals at 72 h and 12 days post-infection. Mixed worm populations recovered at each of these time points were screened under dissecting microscope and sorted into different developmental categories based on size and morphological characteristics. Two to three worms from each group were examined at 100× and measured. Worms recovered from the small intestine at 72 h post infection fell into 3 groups (Additional file 1: Figure S1). Group 1 (length 657.3 ± 13.7 μm) resembled infective L3, with little morphological development in the anterior region. Group 2 worms (776.6 ± 67.4 μm) had developed provisional buccal capsules but had not yet molted, whereas group 3 worms (988.3 ± 102.0 μm) had molted as evidenced by the complete open buccal capsule characteristic of the L4 stage. Subsequently we refer to the groups as 72 h L3, 72 h late L3, and 72 h L4, respectively. Worms collected 12 days post infection were characterized as late L4 if they lacked mature gonads, or as adults if they had mature gonads.

### RNA isolation

Worms collected at 72 h post infection (100–200 worms of each stage) and 12 days post infection (5 worms of each stage) were rinsed with phosphate-buffered saline (PBS) (pH7.4) (Fisher Scientific, Pittsburgh, PA), suspended in 1 mL TRIzol reagent (Invitrogen, Carlsbad, CA) containing 0.5 mm zirconium oxide beads, and homogenized twice for 5 min at maximum speed in a bullet blender (Next Advance Inc, NY). Total RNA was isolated according to the manufacturer’s instructions. Briefly, the homogenate was processed by sequential phase separation and isopropanol precipitation. The RNA pellet was washed with 70% ethanol and resuspended in RNAse-free water. The resulting RNA was treated with RNase-free DNAse I (NEB, Ipswich, MA) to remove any trace of genomic DNA, followed by column purification using RNeasy Mini Kit (Qiagen, Valencia, CA).

### cDNA synthesis

Due to the limited number of worms obtained from hamster small intestines at each time point, cDNA samples were prepared from total RNA isolated from the entire sample using the Super SMART™ PCR cDNA synthesis kit (Clontech, Mountain View, CA). Briefly, first-strand cDNA was synthesized and tailed in the presence of Superscript II reverse transcriptase (Invitrogen, Carslbad, CA), 3′- SMART CDS Primer II A (a modified oligo-dT primer), and SMART II A Oligonucleotide, so that a linker sequence (5′-AAG CAG TGG TAT CAA CGC AGA GT-3′) was added to both 5′- and 3′- ends of the first-strand cDNA. First strand cDNA was purified using NucleoSpin Extract II Columns (Clontech, Mountain View, CA) and further amplified by PCR using primers corresponding to the linker sequence to form double stranded cDNA. The cycling number for amplification was optimized by visual inspection of the exponential phase for each PCR reaction on agarose gels. The double stranded cDNA was purified using NucleoSpin Extract II Column (Clontech, Mountain View, CA) and stored at −20°C until further analysis.

### Quantitative real time PCR (qRT-PCR)

Specific forward (SNR-3-F: 5′-TCT GGC GAA AAC ATC CTG TC-3′, LPP-1-F: 5′-CTG CTG CAA ATA CTC CAA CG-3′) and reverse (SNR-3-R: 5′-TAT CCA GAG ATC CGG AAC TC-3′, LPP-1-R: 5′-CCT CGC AAT TCG TTA GGA AG-3′) primers were designed from *A. caninum snr-3* expressed sequence tag (EXWOO7002HNN28) and *lpp-1* expressed sequence tag (EZAKA7J02F6SNI), respectively, and used to amplify the corresponding fragments of 111 bp and 151 bp. A fragment of 167 bp from the 60S acidic ribosomal protein (60S) was amplified with the primers 60SRP-F (5′-CTG CGT CTG CTG AAG AA-3′) and 60SRP-R (5′-GTC TTG TTG CAT TTC GAG CA-3′) for use as an internal reference gene [34]. All PCR reactions were performed over 40 cycles in triplicate.

Optimal cycling conditions and primer concentration for genes *snr*-3, *lpp-1,* and 60S, were determined using end-point PCR. Amplification efficiencies of the *snr-*3, *lpp-1*, and 60S were determined using standard curve experiments. cDNA templates from different developmental stages were mixed with a specific primer set (*snr-3* or *lpp-1*) and RT^2^ Real-Time™ SYBR Green qPCR Master Mix (SABiosciences, Frederick, MD), and the reaction run in a Bio-Rad CFX96 detection system (BioRad, Hercules, CA) at the optimal conditions described below. The total reaction volume was 25 μL, including 12.5 μL SYBR Green Master Mix, 100 nM forward and 100 nM reverse primers, and 10 ng cDNA. The thermal cycler program was 1 cycle of 95°C for 10 min, followed by 40 cycles of 95°C for 10 s, 62°C for 10 s, and 72°C for 30 s. The same reaction for 60S was set up in parallel.

The threshold cycles (C_T_) were recorded to calculate the ratio of starting concentration or copy number of *snr-*3 (or *lpp-1*) to that of 60S in the original cDNA template, and the relative expression level of *snr*-3 (or *lpp-1*) for each stage-specific cDNA sample was normalized to that in the untreated infective L3s by efficiency adjusted ^ΔΔ^C_T_, a relative quantification method [35,36].

### DNA constructs

The 242 bp amplicon corresponding to the hookworm DAF-16 bound element located within the genomic interval between hookworm *snr-*3 and hookworm *lpp-1* genes was amplified from a pGEM-T EASY construct containing genomic fragment 2.23 (Gao et al., [13]). The specific forward primer (223-FX: 5′- GAT C*TC TAG A*GC AGC TTT ATT CAA GGC GTC-3′, containing restriction site *Xba*I, italicised) and reverse primer (223-RF: 5′-GAT C*CC GGC CGG* CGG AAC TCG TTC ACC AAA C-3′, containing restriction site *Fse*I, underlined), were incubated with the template in a PCR. The cycling conditions were 2 min at 95°C, followed by 35 cycles of 1 min at 95°C, 1 min at 55°C, 1 min at 72°C and a final extension for 6 min at 72°C. The purified amplicon was digested with *Xba*I and *Fse*I, ligated downstream of the luciferase gene on reporter vector pGL4.24 (Promega, Madison, WI) that was cut with the same restriction enzymes to generate pGL4.24-223 construct, and transformed into *Escherichia coli* DH5α competent cells. Similarly, the amplicons from the specific forward primer (223-FXh: 5′-GAT C*CT CGA G*GC AGC TTT ATT CAA GGC GTC, containing restriction site *Xho*I, italicised) and reverse primer (223-RH: GAT C*AA GC T T*CG GAA CTC GTT CAC CAA AC, containing restriction site *Hind*III, italicised), were digested with *Xho*I and *Hind*III, and ligated upstream of the luciferase gene in reporter vector pGL4.24 to generate construct 223-pGL4.24. A similar strategy was employed to clone Fragment 2.23 into a promoter-less luciferase vector, pGL4.12 (Promega, Madison, WI), to generate constructs 223-pGL4.12 with Fragment 2.23 upstream of luciferase, and pGL4.12-223 with Fragment 2.23 downstream of luciferase. The insert sequence of all the reporter constructs was confirmed by DNA sequencing (McLab, South San Francisco, CA).

To determine the importance of the DBE within Fragment 2.23 for *Ac*-DAF-16 mediated transcriptional activities, the DBE deletion mutant constructs were prepared from the reporter constructs mentioned above as templates, using the Site-Directed mutagenesis kit (Stratagene, La Jolla, CA). The resulting mutant plasmids, pGL4.24-Δ223, Δ223-pGL4.24, Δ223-pGL4.12, and pGL4.12-Δ223 were also confirmed by DNA sequencing.

### Cell line and cell culture conditions

The fibroblast cell line NIH3T3 from American Type Culture Collection (ATCC) (Manassas, VA) was used for expression experiments. The cells were maintained in DMEM medium (ATCC, Manassas, VA) containing 10% (v/v) bovine calf serum (BCS) (ATCC, Manassas, VA), 100 I. U. /mL penicillin (Mediatech, Herdon, VA), 100 ug/mL streptomycin (Mediatech, Herdon, VA), 2 mM% L-glutamate (Mediatech, Herdon, VA), at 37°C in an atmosphere of 5% CO_2_. The cells were passaged weekly.

### Transient transfection and reporter assays

NIH3T3 cells grown to 80–90% confluency in 24-well plates with 0.5 mL medium were transfected using Metafectene (Biontex-USA, San Diego, CA) with 500 ng of reporter constructs, 500 ng of pCMV4-DAF-16 construct 2 J [11], and 25 ng of pGL4.74 (Promega, Madison, WI), which is a *Renella* luciferase vector and serves as an internal control. The control vector transfection received 500 ng of reporter constructs, 500 ng of pCMV-TAG4 without an insert (Stratagene, La Jolla, CA), and 25 ng of pGL4.74. To analyze serum response, medium was replaced with 20% (v/v) BCS or serum-free medium at 24 hrs post transfection. Medium was removed 24 hrs later and the cells rinsed with PBS (pH7.4) once.

The relative luciferase activity (firefly luciferase activity divided by the *Renilla* luciferase activity) was determined using Dual Luciferase Assay Reagent (Promega, Madison, WI) and GloMax 96 Microplate Luminometer (Promega, Madison, WI). Briefly, 100 μL of PBS and 100 μL of Dual-Glo™ Luciferase Reagent (reconstituted) were added sequentially to each culture well, mixed and incubated at room temperature for 10 min. Cell lysate (150 μL) was then transferred into a microtiter plate and firefly luciferase signals recorded. Dual-Glo™ Stop-Glo™ (75 μL) was added to microplate wells for 10 min and *Renilla* luciferase signals were recorded. All the assays were performed in triplicate.

Differences between constructs were determined by one-way analysis of variance with Bonferroni’s posttest, and differences between serum and no serum treatments were determined using unpaired student’s *t* test. Differences were considered statistically significant when *p* < 0.05, and the results were expressed as means ± SD. All statistical analysis was performed in GraphPad Prism version 4.01.

## Results

### Gene organization surrounding genomic fragment 2.23

Several *A. caninum* ESTs were identified when 6 kb of genomic DNA surrounding the *Aca*-DAF-16 binding fragment 2.23 was used to search an *A. caninum* EST database available at the Genome Center at Washington University in St. Louis [13]. A BLASTX search revealed that the ESTs represented two neighboring genes in a tail-to-tail orientation with Fragment 2.23 located in the 3′ untranslated region (UTR) of the upstream gene. The translated amino acid sequences from the ESTs EXWOO7002HNN28, EWO9RDQ02JAW57 and EXWOO7002GY2EZ were homologous to *C. elegans snr-3* and that from the ESTs EZAKA7J02GFHML, EZAKA7J01BUK17, EZAKA7J02GG9OI, EZAKA7J02ID2IZ, ETDYEJK02I7C4Z, EXWOO7001CMLKY, EZAKA7J02HCDZS, and EZAKA7J01CP8A8 was homologous to *C. elegans lpp-1* (T28D9.3).

**Figure 1:**
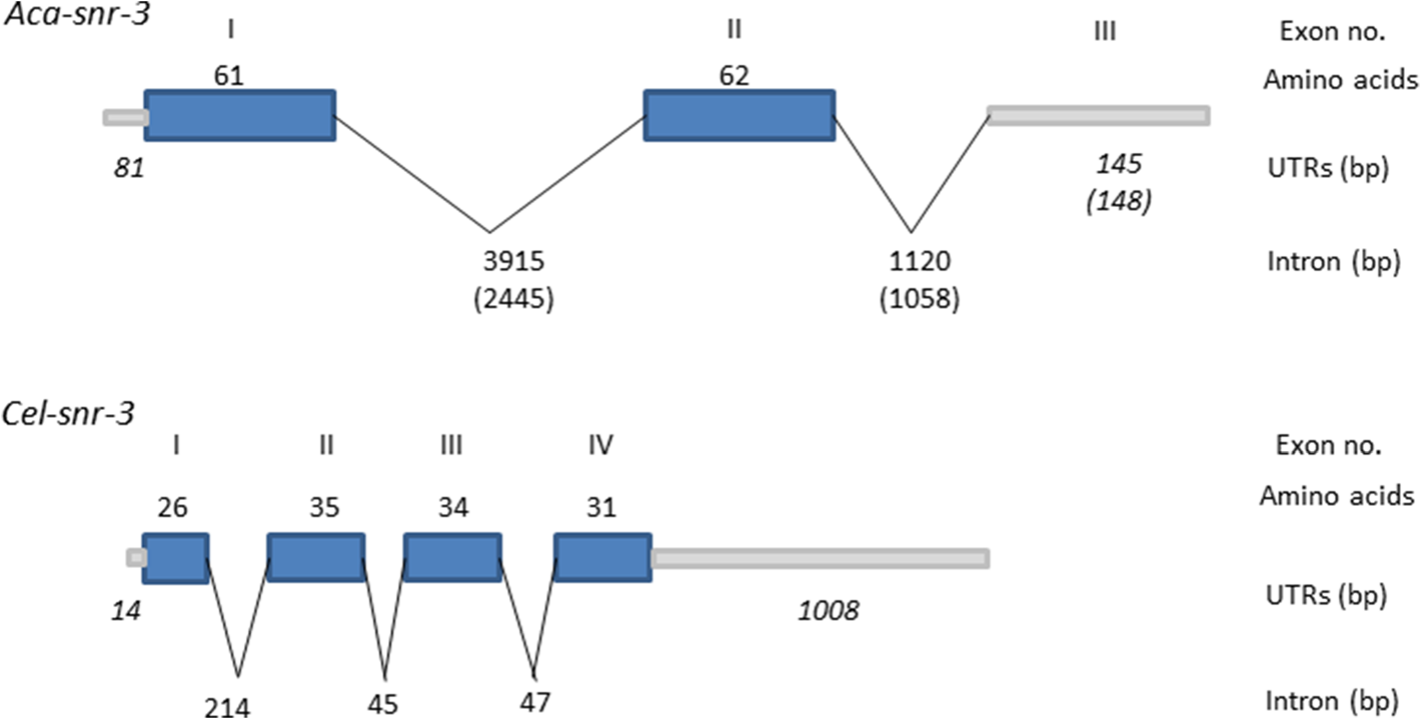
**Comparative gene structure of the hookworm and** ***Caenorhabditis elegans snr-3*** **genes.** Exons are depicted as blue boxes, introns as thin lines, and untranslated regions as gray bars. The length in amino acids of the exons is depicted above the box, and the size of the introns and untranslated regions in base pairs. The numbers in parenthesis represent the lengths for *Ancylostoma ceylanicum snr-3* (EYC42430).

The contig assembled from *snr-3* ESTs was used to search *A. caninum* draft genomic sequence, and a 547 bp isotig (Acan_isotig15549) was identified that contained the full length SNR-3. The isotig contained a 5′ nematode spliced leader [37,38], an open reading frame (ORF) encoded a protein of 123 amino acids, and a 150 bp 3′-untranslated region containing a polyA signal. The isotig was mapped to genomic scaffolds to determine the *Aca-snr-3* gene structure. The *snr-3* gene spans approximately 5.6 kb in the genome and contains 3 exons and 2 introns (Figure 1). The 5′- and 3′- splicing sites of the exon-intron borders conform to the consensus splicing sequences (5′ GT/AC 3′) [39]. The coding region of hookworm *snr*-3 is encoded solely by first two exons. The introns are located between amino acid 61 (Arg) and 62 (Gly), and 2 nt downstream of the stop codon within the 3′ UTR, respectively. Intron I is 3915 bp long and intron II is 1120 bp long. The third exon is 145 bp and contains the complete 3′UTR. Interestingly, the genomic Fragment 2.23 that bound *Aca*-DAF-16 overlaps with both intron II and exon III of hookworm *snr-3*, and the predicted DBE resides at the 3′-end of exon III within the 3′ UTR [13] (Additional file 2: Figure S2). The gene structure of the *A. ceylanicum snr-3* ortholog (EYC42430) is similar to that of *Aca-snr-3*, differing only in the length of the introns and the third exon (Figure 1). The 3′ UTR of *Ace-snr-3* also contains a DBE in the same location as in *Aca-snr-3* (Additional file 2: Figure S2).

**Figure 2:**
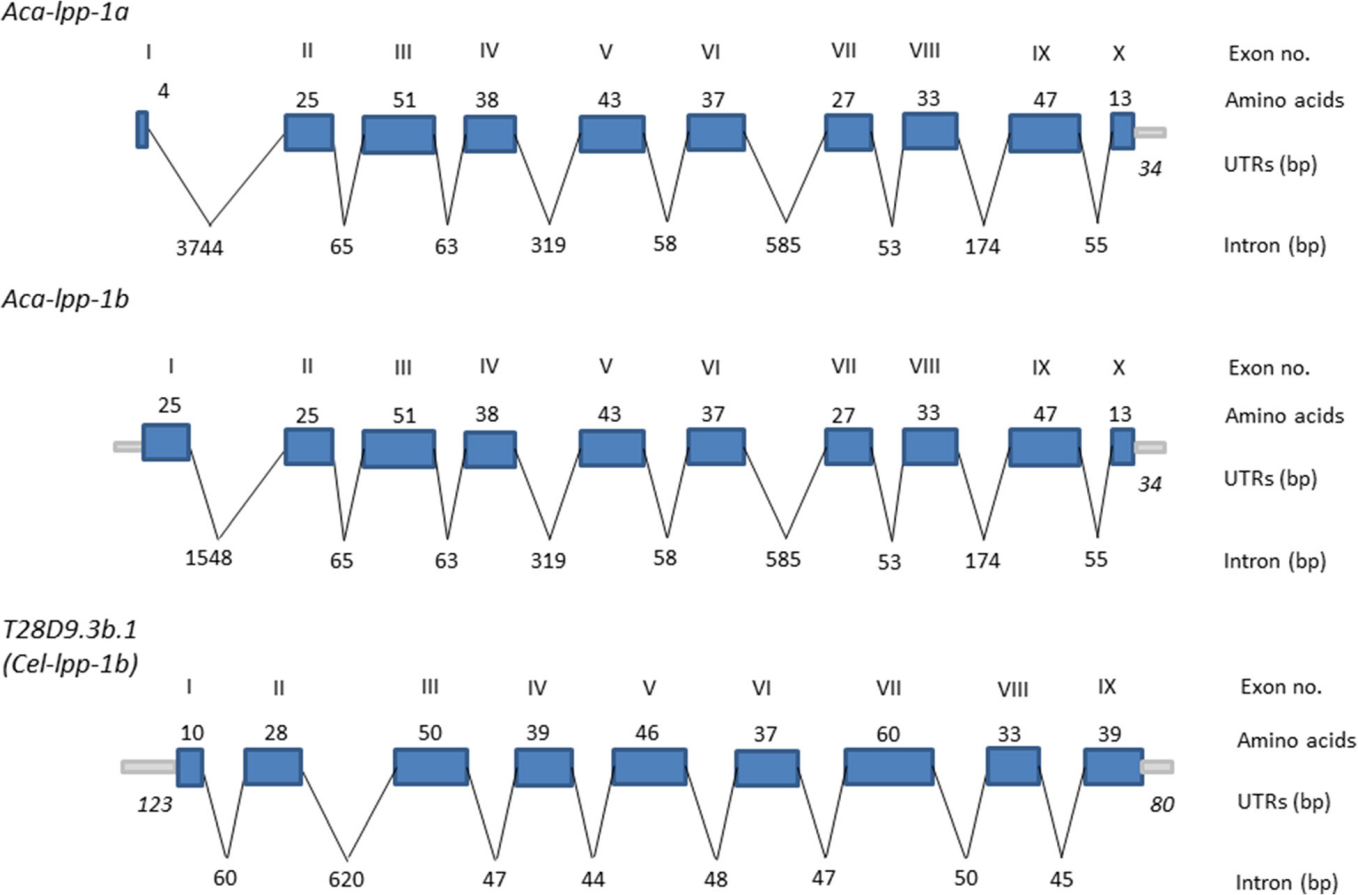
**Comparative gene structure of the** ***Ancylostoma caninum*** **and** ***Caenorhabditis elegans lpp-1*** **genes.** Exons are depicted as blue boxes, introns as thin lines, and untranslated regions as gray bars. The length in amino acids of the exons is depicted above the box, and the size of the introns and untranslated regions in base pairs. The two isoforms of *Aca-snr-3* are shown compared to isoform b of *Cel-lpp-1*.

**Figure 3:**
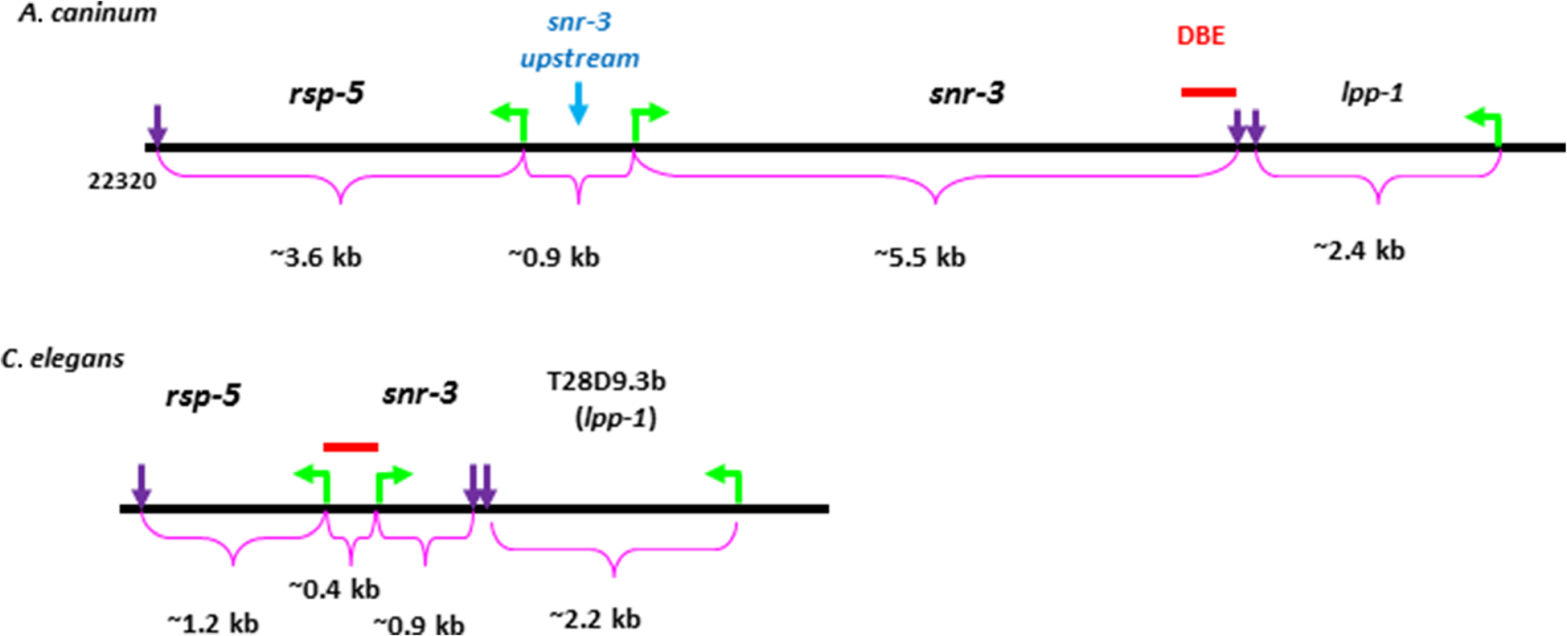
**Genomic neighbors of** ***snr-3*** **.** The diagram depicts the gene arrangement in *A. caninum* and *C. elegans*. The location of the DBE is depicted by a red bar. The DBE located upstream of *Cel-snr-3* was reported in Wormbase.

Downstream from *snr-3* is a gene encoding *lpp-1* (Figure 2). The genes are arranged in a tail-to-tail arrangement on opposite DNA strands, with 27 bp separating the 3′ ends (Figure 3). Searching draft *A. caninum* genomic sequence with the DNA sequence assembled from *lpp-1* ESTs identified Acan_isotig07031 containing a full length ORF encoding a protein of 318 amino acids, with a 35 bp 5′ UTR and a 35 bp 3′-untranslated region containing a polyA tail signal. We used the predicted LPP-1 amino acid sequence to search the *A. caninum* draft gene set and identified a second, longer isoform that encoded a 339 amino acid ORF. A truncated EST (EZAKA7J01CP8A8) that maps to the first exon supports this model. The 2 forms appear to be splice variants, as only the first exons differ. Exon 1 of the shorter form (isoform a) encodes 4 amino acids, whereas exon 1 of isoform b encodes 25 amino acids. The entire *lpp-1* gene spans over 6.0 kb and contains 10 exons and 9 introns. Intron 1 is the longest, spanning 3.7 kb in isoform a, and 1548 in isoform b. The remaining introns range from 53 to 319 bp (Figure 2). The 5′- and 3′- splicing sites of the exon-intron borders of *lpp-1* also conform to the consensus splicing sequence (5′ GT/AC 3′) [39].

Subsequent to our identification of *Aca-lpp-1*, genomic and transcriptomic sequence data from *A. ceylanicum* was deposited in Genbank (PRJNA231479, PRJNA231490). Using the *Aca*-LPP-1 protein sequence to search Genbank, we identified 7 predicted splice isoforms of *Ace-lpp-1* (Additional file 3: Figure S3). The two longest isoforms (EYC42428 and EYC42427) contained 10 exons like *Aca-lpp-1*. However, the first exon of EYC42427 was truncated to the final 4 amino acids of EYC42428 exon 1. Unlike *Ace*-LPP-1, the N terminal amino acids of the *Aca*-LPP-1 isoforms are derived from different exons rather than truncation of the same exon. Isoforms EYC42424, EYC42425, and EYC42426 differ from EYC42427 at the C-terminus, with EYC42425 lacking exon 10, EYC42424 lacking exons 7–10, and EYC42426 having an alternate exon 7 and lacking exons 8–10. Finally, EYC42423 and EYC42429 are composed of exons 7–9 and 7–10 respectively. In both cases, exon 7 is truncated by 6 amino acids, and in EYC42423, exon 9 is lengthened by 13 amino acids (Additional file 3: Figure S3). There are also 5 isoforms of T28D9.3, the *C. elegans lpp-1* gene, in Wormbase (wormbase.org). We were unable to identify more than 2 isoforms in the available *A. caninum* genomic and transcriptomic sequence, and only a single isoform (ETN74806) was found in the genome of the related hookworm *Necator americanus* (PRJNA72135) [9].

The hookworm *snr-3* and *lpp-1* genes are organized differently from the *C. elegans* corresponding genes. In *C. elegans*, *snr-3* is encoded in 4 exons, with the 3 introns of 214, 45, and 47 nt, whereas the hookworm *snr-3* genes are encoded by 3 exons separated by 2 very long introns. Interestingly, the introns in *Ace-snr-3* are shorter than those in *Aca-snr-3*, although still much longer than those in *C. elegans* (Figure 1) The *lpp-1* genes are similar, with those of hookworm having more exons (10 vs 9) and longer introns (Figure 2). Despite the differences in gene structure, however, the gene arrangement at this locus is the same in hookworms and *C. elegans* (Figure 3). The *snr-3* and *lpp-1* genes are on opposite strands in a tail-to-tail arrangement in both species, separated by 27 nt in *A. caninum* and *A. ceylanicum*. Additionally, upstream of *snr-3* on the opposite strand in the hookworm and *C. elegans* genomes is the *rsp-5* gene that encodes for the ortholog of the vertebrate SC35 splicing factor [40,41] (Figure 3). We did not further analyze the *rsp-5* gene. While the gene order is conserved, there is no DBE located at the 3′ end of the *Cel-snr-3* gene. However, a DAF-16 binding site was identified by ChIP in the region between the *snr-3* and *rsp-5* genes in *C. elegans* (Figure 3) (http://www.wormbase.org). We could not identify a DBE in the corresponding region in the hookworm genome (i.e. upstream of *snr-3*).

### Functional domains and evolutionary relationships of *snr-3* and *lpp-1*

**Figure 4:**
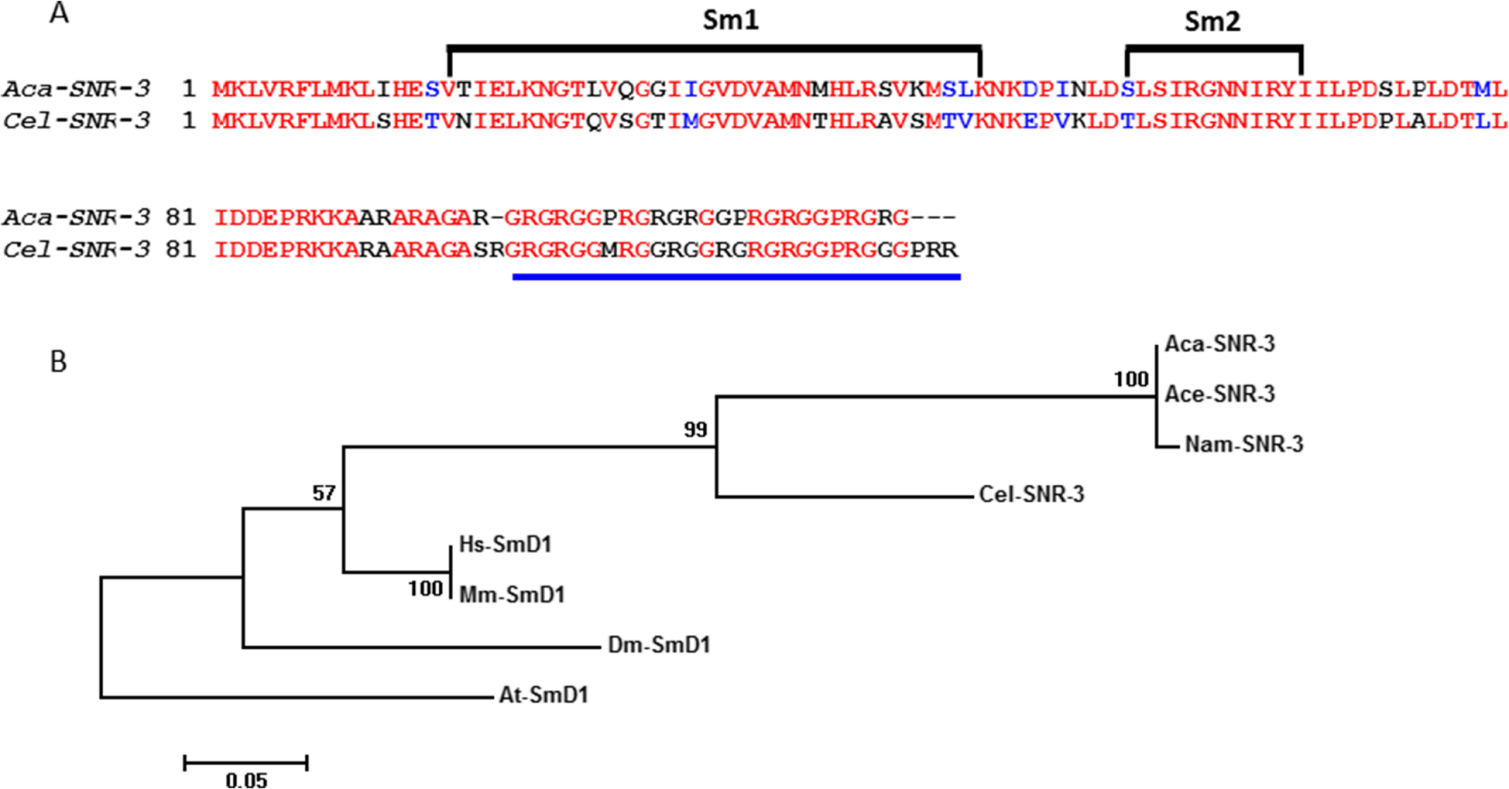
***Aca-snr-3*** **encodes the hookworm core Sm protein D1 (Sm-D1). A**. *Aca*-SNR-3 translated amino acid sequence aligned with its ortholog *Cel*-SNR-3. Black brackets represent the conserved Sm domains involved in protein-protein interactions. The underlined region is the hallmark Gly-Arg repeats. **B**. Maximum likelihood tree showing relationship of *Aca*-SNR-3 to other Sm-D1 proteins. The percentage of trees in which the associated taxa clustered together is shown next to the branches. The *A. thaliana* sequence was designated as the out group. Accession numbers: Ace, *A. ceylanicum*, EYC42430; Nam, *Necator americanus*, ETN74807; Cel, *Caenorhabditis elegans*, NP_495306; At-SmD1 *Arabidopsis thaliana*, NP_187416; Mm-SmD1 *Mus musculus*, EDL22993; Hs-Sm D1 *Homo sapiens*, NP_008869; Dm-SmD1 *Drosophila melanogaster*, NP_524774.

As shown in Figure 4A, SNR-3/snRNP D1 proteins including *Aca*-SNR-3 share two characteristic Sm protein motifs, Sm1 and Sm2, as well as a conserved C-terminal structural domain, a Gly-Arg repeat. Sm motifs are essential for formation of a closed ring of the Sm complex [19,20,42]. The Gly-Arg repeat is expected to have strong binding affinity for nucleic acids due to the relative abundance of basic residues. Additionally, it represents one of the Sm-D immunoreactive determinants and shows significant sequence similarity with a region in the Epstein-Barr nuclear antigen-1 that is a non-Sm protein [43,44].

A BLASTP search of the non-redundant Genbank database using the conceptual translation of *Aca*-SNR-3 revealed high homology with SNR-3/snRNP D1 proteins from organisms as diverse as *C. elegans* (69% identity and 78% positive over 88 amino acids), *Drosophila melanogaster* (73% identity and 89% positive over 111 amino acids), *Arabidopsis thaliana* (64% identity and 79% positive over 95 amino acids), *Mus musculus* (67% identity and 82% positive over 96 amino acids), *Homo sapiens* (67% identity and 82% positive over 96 amino acids), and yeast *Schizosaccharomyces japonicus* (57% identity and 78% positive over 94 amino acids), suggesting a high level of evolutionary conservation. *Aca*-SNR-3 was identical to *Ace*-SNR-3 (EYC42430), and 99% identical to *Nam*-SNR-3 (ETN74807). As expected, phylogenetic analysis using aligned SNR-3 of these species showed that the hookworms form a sister group with *C. elegans*, and are more distant from the other taxa (Figure 4B).

**Figure 5:**
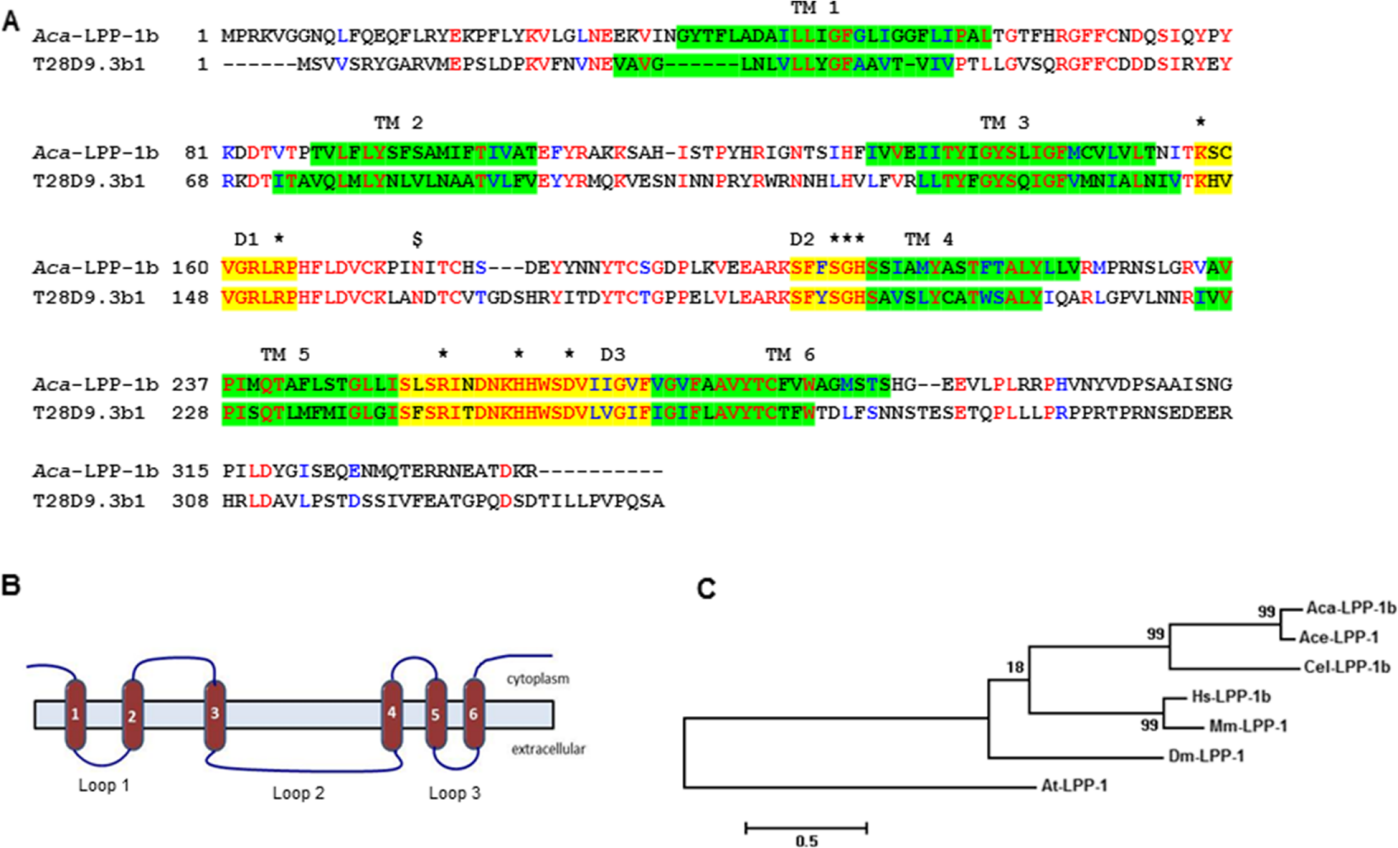
***Aca-lpp-1*** **encodes a lipid phosphate phosphatase. A**. *Aca*-LPP-1b translated amino acid sequence aligned with its *C. elegans* ortholog T28D9.3b1. Identical residues are in red, and conserved residues in blue. The phosphatase active site domains are highlighted in yellow, and the conserved residues required for activity are marked with an asterisk. The transmembrane domains are highlighted in green, and the conserved N-linked glycosylation site is marked by a dollar sign. **B**. Structure of LPP-1 showing transmembrane domains. Active site residues are found on extracellular loops 2 and 3. **C**. Maximum likelihood tree showing relationship of *Aca*-LPP-1 to other LPP-1 proteins. The percentage of trees in which the associated taxa clustered together is shown next to the branches. The *A. thaliana* sequence was designated as the out group. Ace, EYC42428.1; Cel, NP_001022378; At AAM63082; Mm, AAH61161; Hs, EAW54922; Dm, NP_649394.

Lipid phosphate phosphatase (LPPs) are cell surface, N-glycosylated integral membrane proteins that catalyze the hydrolysis of lipid phosphate mono-esters [45,46]. The active site amino acids are exposed on the exterior of the membrane, indicating that LPP-1 is an exophosphatase. *Aca*-LPP-1 is homologous to the Mg^2+^- independent phosphatidic acid phosphatases (PAP-2). Because of its relatively catholic lipid phosphate substrate specificity, PAP-2 was renamed lipid phosphate phosphatase [45,47]. LPPs belong to the wunen subfamily of membrane associated PAPs, and have three conserved active site domains and 6 membrane-spanning regions of 20 or 23 hydrophobic amino acid residues, all of which are conserved in *Aca*-LPP-1 [47,48] (Figure 5A and B). Unlike *Aca*-SNR-3, *Aca*-LPP-1 displayed relatively low homology (around 40% or below) with the corresponding peptides from other species. Phylogenetic analysis using aligned LPP-1 sequences from different species grouped hookworm LPP-1 most closely with *C. elegans* LPP-1, and more distantly with *A. thaliana* ones (Figure 5C).

### Developmental expression patterns of *snr-3* and *lpp-1* post infection

To determine the expression patterns of *snr-*3 and *lpp-1* during hookworm parasitic development, cDNAs were synthesized from mRNAs of five categories of *A. ceylanicum* worms representing the distinct developmental stages recovered from infected hamsters. *A. ceylanicum* in Golden Syrian Hamsters has been a useful model to mimic the sequelae and physical symptoms that occur during human hookworm infection [49-51]. Its ability to infect a small rodent host allows relatively tractable *in vivo* time-series experimentation.

Since it is almost impossible to get a synchronized population of the *A. ceylanicum* worms (or any hookworms) after they enter the host, recovered worms were categorized based on appearance under a dissecting microscope. Examination of sample worms from each category under higher magnification indicated that the five categories roughly represented L3, late L3, L4, late L4 and adult stages, respectively.

**Figure 6:**
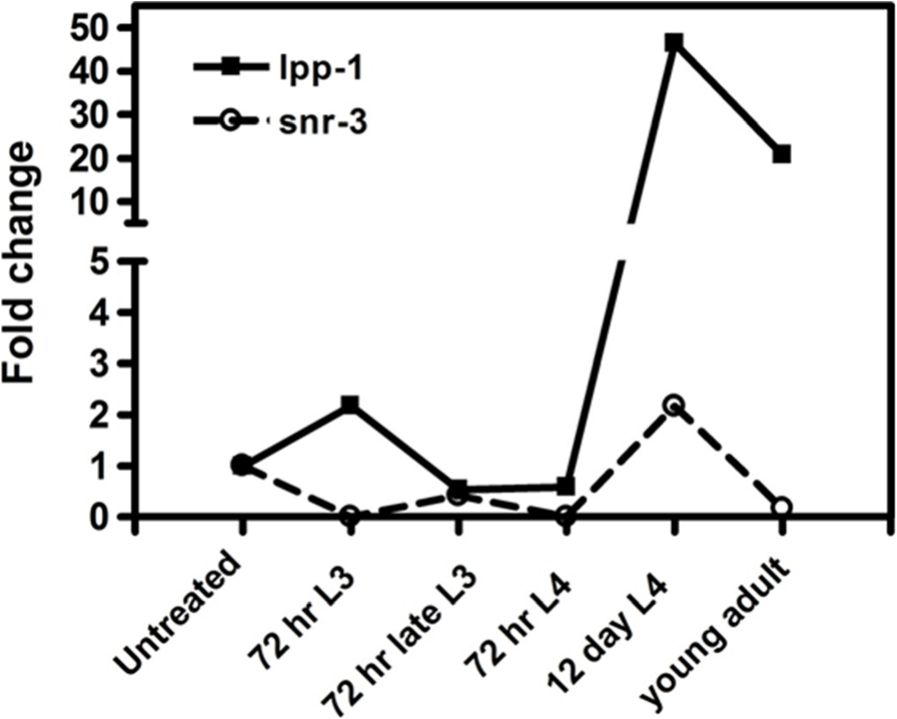
**Transcription of hookworm** ***snr-3*** **and** ***lpp-1*** **during parasitic development.** Developing *A. ceylanicum* were recovered from infected hamsters at 72 h and 12 days post-infection and separated into life cycle stage for RNA isolation. The fold change relative to the infective L3 stage and normalized to the 60 s ribosomal protein reference gene is shown.

qRT-PCR using pre-amplified double stranded cDNA indicated that, other than a slight increase in *lpp-1* expression in 72 h L3, the transcription of both *snr-3* and *lpp-1* stayed low or undetectable in all of the early post infection parasitic stages (Figure 6). However at 12 days post infection, levels of these two genes were up-regulated in the late L4 stage. *Ace-snr-3* transcription increased 2-fold relative to infective L3, whereas *Ace-lpp-1* transcript levels increased nearly 50-fold. *Ace-snr-3* transcription was down regulated severely in early adults, while *Ace-lpp-1* transcript levels fell somewhat, but remained 20-fold higher than untreated infective L3 (Figure 6). The elevated expression of *lpp-1* in the 12 day L4 stage suggests high levels of phosphatase activity, perhaps associated with signaling events accompanying the upcoming molt to the adult and concomitant gonad development. The increases in *snr-3* transcription in 12 day L4s may also represent gene splicing in preparation for this molt.

### Genomic fragment downstream of hookworm *snr-3* coding region was implicated in the regulation of *Aca-snr-3* gene expression

**Figure 7:**
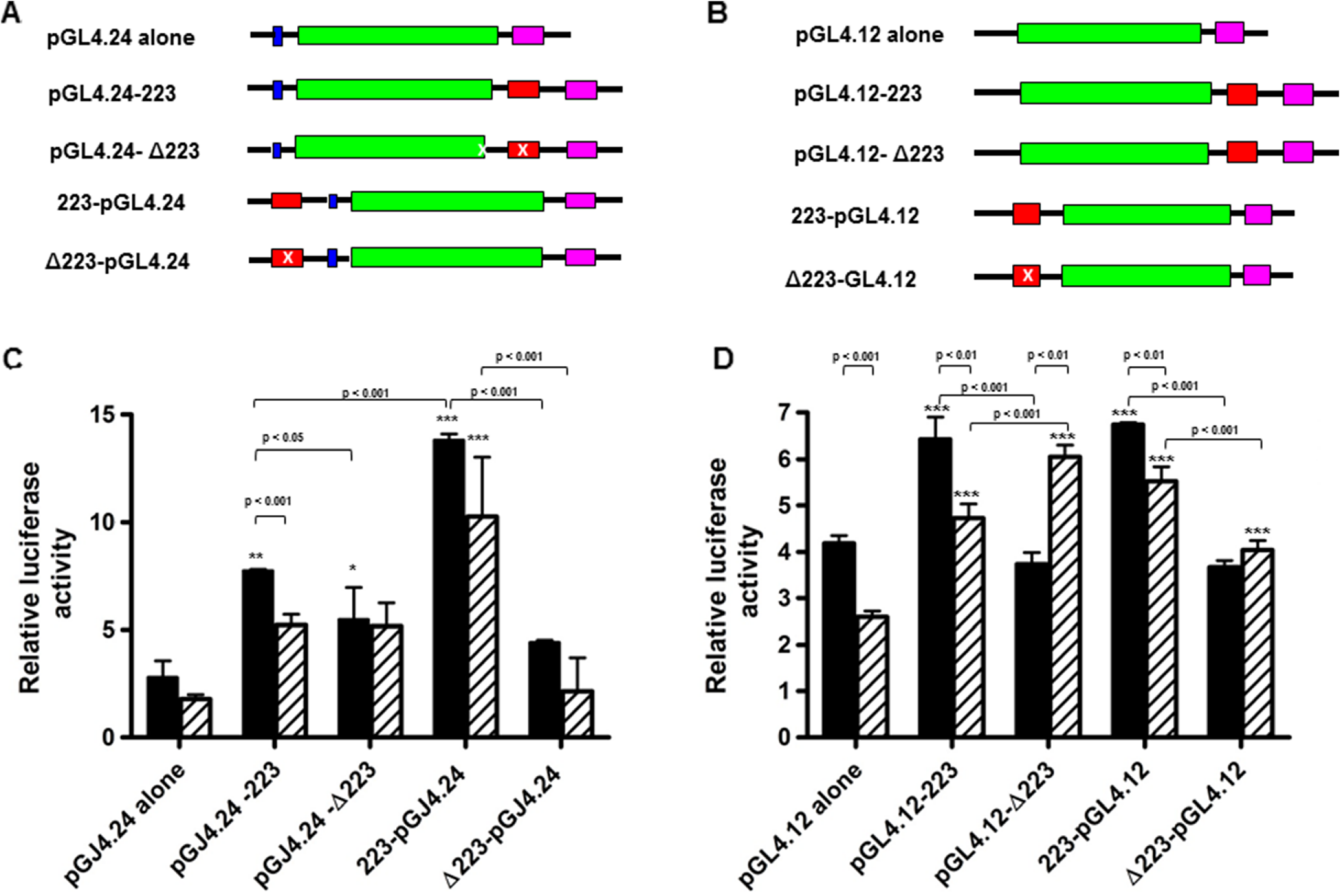
***Aca*** **-DAF-16 drives expression of luciferase from Fragment 2.23. A**. Fragment 2.23 (red) was cloned into different positions relative to reporter luciferase gene (green) in vector pGL4.24. Pink boxes represent the polyadenylation site of the vector, and blue boxes represent the minimal promoter. DBE deletion mutants are represented by an “x” in the red box. **B**. Fragment 2.23 was cloned into different positions relative to reporter luciferase gene in vector pGL4.12. **C**. Relative luciferase expression in NIH3T3 cells co-transfected with the pGL4.24 and pCMV4-DAF-16 constructs. Solid bars, no serum; striped bars, 20% serum. Results are normalized to empty vector control transfection. **D**. Relative luciferase expression in NIH3T3 cells co-transfected with the pGL4.12 and pCMV4-DAF-16 constructs. Significant differences between luciferase expression of Fragment 2.23 constructs and their appropriate plasmid alone control are denoted by asterisks (*p < 0.05, **p < 0.01, ***p < 0.001). Differences between other comparisons are shown with a bracket and p value. Differences within groups (serum or no serum) were determined by one-way ANOVA with Bonferroni’s Multiple Comparison post-test, and differences between serum and no serum were determined by unpaired t-test using GraphPad Prism.

The 242 bp genomic Fragment 2.23 that spans intron II/ exon III of the hookworm *snr-3* gene displayed strong *in vitro* binding to the DBD of *Aca*-DAF-16, and a DBE was identified in its 3′-terminus [13]. Fragment 2.23 is closely linked to two neighboring genes, suggesting that it might regulate gene expression of *snr-3* and *lpp-1*. To test this hypothesis, genomic Fragment 2.23 was cloned downstream and upstream of the firefly luciferase gene in pGL4.24 containing a minimal promoter, or in the promoter-less luciferase vector pGL4.12 (Figure 7A and B).

The resulting luciferase constructs, designated as 223-pGL4.24, pGL4.24-223, 223-pGL4.12, and pGL4.12-223, were analyzed for their effects on reporter expression by transient co-transfection with *Aca*-DAF-16 in NIH3T3 cells. As seen in Figure 7C, in the absence of serum both constructs 223-pGL4.24 and pGL4.24-223 significantly increased luciferase expression in the presence of *Ac*-DAF-16 compared with empty pGL4.24 vector transfection control (*p* < 0.01), indicating that the regulatory activity of Fragment 2.23 is independent of its position (upstream or downstream) relative to luciferase gene. However, this effect was greater when Fragment 2.23 resided upstream of the luciferase gene, as there was two-fold higher relative luciferase activity for construct 223-pGL4.24 than for construct pGL4.24-223, suggesting that the interaction mode between Fragment 2.23 and DAF-16 may differ depending on the relative location of this fragment to the corresponding gene. Deletion of the DBE in Fragment 2.23 significantly reduced luciferase expression in both constructs, and decreased expression to control levels in Δ223-pGL4.24 containing cells (Figure 7C).

In constructs without a promoter (223-pGL4.12 and pGL4.12-223), Fragment 2.23 had a similar but less pronounced effect on luciferase expression in the presence of *Ac*-DAF-16 without serum (Figure 7D). Unlike pGL4.24 constructs, Fragment 2.23 increased luciferase expression to similar levels in either the upstream or downstream position in the absence of a minimal promoter. Deletion of the DBE in Fragment 2.23 reduced luciferase expression to control levels regardless of whether the fragment was upstream or downstream of the luciferase gene in the absence of serum.

DAF-16 is negatively regulated by the ILS pathway [52-54]. The DBE-mediated transcriptional activity of DAF-16 in response to serum stimulation was reported previously using a mammalian cell culture system [11,55]. Therefore we were interested in determining whether serum had similar effects on the interaction of *Ac*-DAF-16 and Fragment 2.23. In constructs containing the minimal promoter and incubated with 20% serum, Fragment 2.23 failed to significantly increase luciferase expression when located downstream of the luciferase gene, but increased expression to similar levels as without serum when located upstream (Figure 7C). In the constructs lacking promoters, Fragment 2.23 increased expression relative to the control in either location relative to the luciferase gene, although to generally lower levels than that in the absence of serum (Figure 7D). One exception is in the construct containing DBE deletion mutant Fragment 2.23 downstream of the luciferase gene, where the expression level is higher than the wild type Fragment 2.23 when incubated in 20% serum. Aside from this enigmatic response, the inclusion of serum had little effect on expression in this cell system.

## Discussion

The forkhead transcription factor DAF-16 has emerged as a master regulator for many important biological processes since it was first identified in *C. elegans* [56-58]. Accumulating studies indicate that DAF-16 mediates its functions by regulating the expression of different target genes [16,17,59-61]. The presence of DAF-16 orthologs has been reported from several parasitic nematodes in the past decade [11,62,63]. Parasitologists are particularly interested in DAF-16 and how it regulates its downstream gene network, since DAF-16 plays critical roles in dauer formation and exit in *C. elegans*, which has been adopted as a model for studying the infectious process of parasitic nematodes. The present study described the first characterization of putative hookworm DAF-16 target genes, *Ac-snr-3* and *Ac-lpp-1*.

*Ac-snr-3* encodes a core Sm protein, Sm-D1, present in snRNP particles, and *Ac-lpp-1* encodes a lipid phosphate phosphohydrolase. Sequence analysis indicated that the deduced amino acid sequences of both *Ac*-SNR-3 and *Ac*-LPP-1 shared the most similarities with their counterparts from *C. elegans*. However, the corresponding gene structures are quite different between these two species even though they belong to the same phylogenetic clade [64]. The *C. elegans snr-3* and *lpp-1* genes are shorter in overall length and have much shorter introns. This may reflect the difference in genome size between hookworms (~347 Mb) and *C. elegans* (100 Mb) [65,66]. Further analysis of gene structure in hookworms will provide additional insights.

The hookworm SNR-3 protein sequence contained all the important hallmarks of the Sm superfamily including the conserved Sm-motifs and invariable amino acids involved in hydrophobic interactions, salt bridge formation, and hydrogen bonding [20]. Unlike in the mammalian and yeast systems, none of the protein components of the splicing machinery have been identified in hookworm. Nematodes are unique in that their expression of protein-coding genes requires *trans*-splicing in addition to *cis*-splicing. In *C. elegans*, about 70% of mRNAs are *trans*-spliced to one of two 22 nucleotide spliced leaders (SLs) [67]. The processing events of *trans*-splicing are closely related to *cis*-splicing [68]. Hookworm presumably employs a similar gene splicing mechanism, as SL sequences have been identified from *A. caninum* mRNAs [38].

LPPs are cell surface, N-glycosylated integral membrane proteins that catalyze the hydrolysis of lipid phosphate monesters [45,47]. The active site amino acids are located on the extracellular loops (exofacial), indicating that LPP-1 is an exophosphatase [23,47]. Hookworm LPP-1 was predicted to have the featured transmembrane regions and the conserved amino acids involved in the enzyme activities [69]. Similar to SNR-3, it is the first enzyme relevant to phospholipid metabolism identified in hookworms. LPPs regulate lipid signaling by altering the concentrations of phosphorylated and dephosphorylated lipids [23]. The direct involvement of PIP3 as a second messenger in DAF-16 mediated insulin-like signaling pathway [70] indicates that a balanced phospholipid pool is critical for the worm and highlights the significance of LPP-1 function.

Since the expression changes of Sm proteins and LPP-1 during worm development are potentially important in pre-mRNA processing and phospholipid metabolism, respectively, we generated transcriptional profiles of hookworm *snr-3* and *lpp-1* across several post-infection stages using qRT-PCR data. Unexpectedly, hookworm *snr-*3 transcription was turned off and *lpp-1* transcription was turned on immediately after the infective L3 entered the host small intestine. Microscopic examination under high magnification showed that the morphology and behavior of the L3s at this stage was almost identical to those of the infective L3s. However within the worm, dramatic biochemical changes have been triggered in preparation for resumption of parasitic development once host signals are received, and *snr*-3 and *lpp-1* represent two early responding genes that are regulated by those signals. Specifically, SNR-3 is postulated to be indispensable in all hookworm developmental stages, and the extreme low level of *snr-3* transcripts in host L3s suggests that sufficient SNR-3 proteins for post-infection development may have been stored in the L3 for exit from the arrested state and the subsequence development. However, the possibility that different, as yet unidentified, isoforms of hookworm *snr-3* might be transcribed in early post-infection stages cannot be excluded. The transcription of both hookworm *snr-*3 and *lpp-1* peaked at the late L4 stage, suggesting active splicing and dephosphorylation events were required for the worm’s maturation. LPP-1 levels peaked at nearly 50-fold up-regulation in 12 day L4, suggesting a role of lipid signaling in development of the gonads and maturation to the adult reproductive stage. Since the data were obtained from a mixed population of whole worms, the transcriptional increase at this stage in specific sexes, tissues or cells might be even greater.

The distinct temporal transcription profiles of hookworm *snr*-3 and *lpp-1* after infection indicated that these two genes were subject to tight and different regulations. The difference in the expression patterns of these two genes in host-derived parasitic L3s suggested the opposing roles DAF-16 could play during the early infective stage. A 242 genomic fragment (Fragment 2.23) containing a reverse DBE (5′-AACAAATA-3′) at the 3′-terminus of the *Ac-snr*-3 gene was found to strongly interact with *Ac-*DAF*-*16 DBD in binding assays, as evidenced by its high frequency in our isolated genomic DNA [13]. We hypothesized that this genomic fragment acts as a *cis*-regulatory element affecting expression of a surrounding gene. Here we co-transfected mammalian cells with *Ac-DAF*-16 and reporter constructs containing Fragment 2.23 upstream or downstream of the luciferase gene and measured the relative luciferase activities. In the presence of a minimal promoter, Fragment 2.23 was able to drive luciferase expression to high levels, especially when located upstream of the luciferase gene, suggesting it interacts with the minimal promoter to enhance expression. More interesting is the ability of Fragment 2.23 to drive expression of luciferase in the absence of a vector encoded promoter elements, regardless of its location relative to the luciferase gene. The ability to drive expression from the downstream location, with or without a minimal promoter, indicates that Fragment 2.23 contains activity that enhances baseline expression of luciferase in this expression system. Since the transcriptional activity does not depend on its relative position to the reporter gene, it suggests that Fragment 2.23 might simultaneously influence the expression of both upstream and downstream genes in the genomic background, acting as either a promoter or enhancer. Shared *cis*-regulatory elements are not unusual in strongly correlated gene pairs or gene clusters and have been implicated in coordinating gene expression [71-74]. Further characterization of neighboring genes will undoubtedly unravel more information about the function of the regulatory elements within this genomic fragment. It is also worth noting that the mammalian cell culture system provides a simplified physiological setting for activity studies of gene regulatory elements, and the observed positive regulation mediated by Fragment 2.23 using this system might not be indicative of the true regulatory direction in the physiological setting of the hookworm.

To investigate the role of the DBE element within the Fragment 2.23 on the expression of *Ac*-DAF-16 candidate gene targets, the constructs containing deleted DBEs were generated. In the absence of serum, deletion of the DBE abrogated the transcriptional activity of Fragment 2.23 regardless of its location relative to the luciferase gene. This suggests that DAF-16 binding is required for the transcriptional activity of Fragment 2.23. We observed that the deletion had a greater effect when Fragment 2.23 was located upstream of the reporter gene and the minimal promoter was present. A possible explanation is that a different set of protein co-factors were involved in the interaction between *Ac*-DAF-16 and Fragment 2.23 when the location of Fragment 2.23 relative to the target gene was different.

Previous findings showed that serum depletion greatly up-regulated reporter gene expression mediated by the consensus DBE, which was consistent with the conclusions that the critical factor for the determination of transcriptional activity of DAF-16 orthologues was their cellular location [11]. When similar experiments were performed in the current study, Fragment 2.23 was not responsive to serum stimulation. These results were not surprising since Fragment 2.23 is a much longer DNA sequence compared with the 8-bp consensus DBE, and scanning the sequence for transcription factor binding sites showed that it potentially contains more complicated regulatory elements. Under such circumstances, Fragment 2.23 might be capable of recruiting additional protein factors that modify the interaction between Fragment 2.23 and *Ac*-DAF-16. For example, a well-documented forkhead protein co-factor is C/EBPβ (CCAAT-enhancer-binding protein beta), and its binding motif is highly over-represented in Fragment 2.23 [75-77]. Transcriptional stimulation resulting from interaction between FOXO and C/EBPβ has been demonstrated to be greater than additive [78,79]. Another regulatory element found in Fragment 2.23 is HSF-1 (Heat Shock Factor-1) binding element (HSE). HSEs co-exist with DAF-16 binding elements in several *C. elegans* promoters, where together they activate expression of specific genes such as the *shsp* (Small Heat-Shock Proteins) [80]. Our results suggest that using a native genomic fragment might help to identify new co-factors interacting with *Ac*-DAF-16 and provide insights into the mechanisms that are relevant to how signaling events involving DAF-16 are integrated with the transcriptional machinery.

The function of a DBE site at the 3′ end of the *snr-3* gene and its role in the expression of *snr-3* or the downstream gene *lpp-1* is unknown. Our data indicates that Fragment 2.23 can function as an enhancer or promoter in *cis*, at least an *in vitro* based cell culture system, and that the DAF-16 dependent activity requires the DBE sequence. The tail-to-tail arrangement of *snr-3 and lpp-1* in the genome places the DBE distal to the upstream regulatory sequences of both genes. This enigmatic location outside a traditional promoter suggests that the DBE may act at a distance to influence transcription. Further investigations are required to test this hypothesis.

## Conclusions

In conclusion, we report the gene structure of hookworm DAF-16 putative targets, *Aca*-*snr*-3 and *Aca-lpp-1* which were identified by genome wide screening for *Ac*-DAF-16 binding elements. *Aca*-*snr*-3 encodes a core small nuclear ribonucleoprotein and the predicted amino acid sequence shares considerable homology with other Sm-D1 proteins. *Aca-lpp-1* encodes a lipid phosphate phosphohydrolase. Messenger RNA levels of both genes were tightly regulated after host entry and peaked at the late L4 stage, suggesting a potential role in L4 development. The 3′-terminal genomic fragment of this gene displayed *Aca*-DAF-16-dependent *cis*-regulatory activity. Further investigations of the regulation of hookworm *snr*-3 and *lpp-1* expression will illuminate the physiological role of *Aca*-DAF-16.

## Additional files

Additional file 1: Figure S1. Representative *Ancylostoma ceylanicum* stages collected at 72 h post infection. A. Parasitic L3 stage. Note the lack of significant morphological remodeling in the anterior. B. Late parasitic L3 stage. Note the provisional buccal capsule longitudinally bisected by the cuticle lining of the former buccal cavity (arrow). C. Parasitic L4 stage. Note the complete buccal capsule characteristic of this stage (arrow). Additional file 2: Figure S2. Map of the *Aca-snr-3* gene showing the location of Fragment 2.23 and the Daf-16 binding element. Exons are indicated by boxes, with coding regions in green and untranslated regions in aqua. Introns are indicated as lines. The red bar shows the approximate location of Fragment 2.23, and the inset shows its DNA sequence. The 3′ sequence of intron 2 is in italicized lowercase text, exon 3 sequence is in blue uppercase text, and the DBE in red text. The 3′ splice sequence of intron 2 is underlined, the polyadenylation signal sequence is in pink text, and the site of polyA tail addition is boxed. Numbering is from the original contig sequence. Additional file 3: Figure S3. Predicted isoforms of *Ace*-LPP-1. Isoforms sequences, indicated by accession number, were obtained from Genbank and mapped to *A. ceylanicum* draft genomic contigs to determine gene structure. Similar exons are shown in the same color. The number of amino acids is shown above each exon. The intron length in base pairs is indicated below each intron. The location of the primers used for qPCR is indicated by arrowheads.

## Abbreviations

BCS: Bovine calf serum
DBD: DNA binding domain
DBE: DAF-16 family binding element
EST: Expressed sequence tag
HSE: Heat shock element
LPP: Lipid phosphate phosphatase
ORF: Open reading frame
snRNP: Small nuclear ribonucleoproteins
UTR: Untranslated region
