# Supplementary figures and images for: Two potential hookworm DAF-16 target genes, SNR-3 and LPP-1: gene structure, expression profile, and implications of a cis-regulatory element in the regulation of gene expression
Source: Parasit Vectors. 2015 Jan 8;8:14. doi: 10.1186/s13071-014-0609-0 (PMC4298947; doi:10.1186/s13071-014-0609-0)

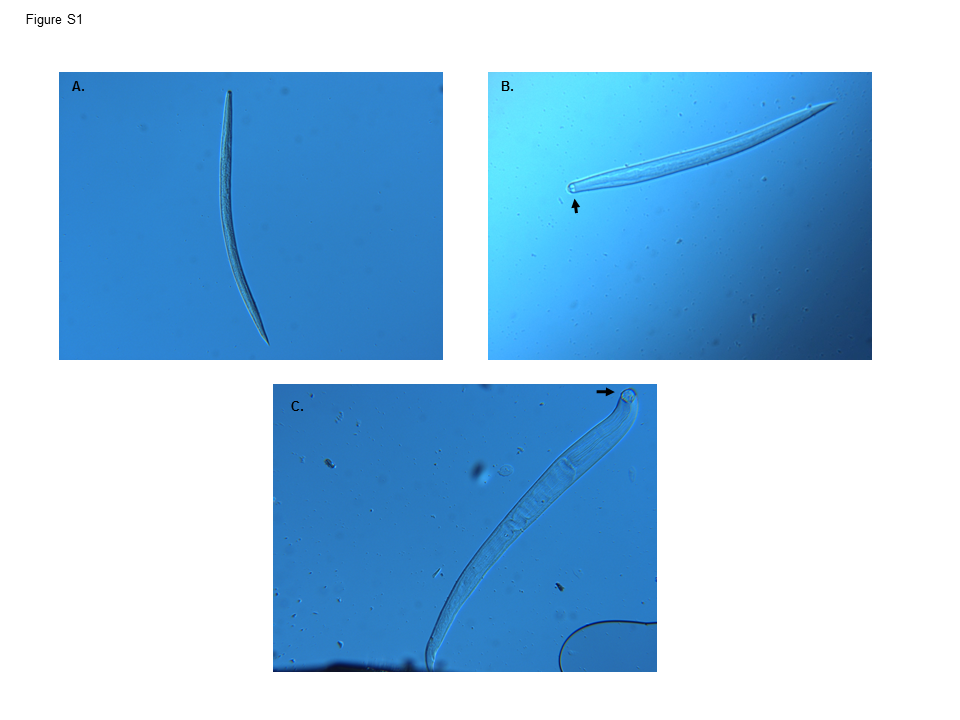

Supplement: Additional file 1: Figure S1. — Representative Ancylostoma ceylanicum stages collected at 72 h post infection. A. Parasitic L3 stage. Note the lack of significant morphological remodeling in the anterior. B. Late parasitic L3 stage. Note the provisional buccal capsule longitudinally bisected by the cuticle lining of the former buccal cavity (arrow). C. Parasitic L4 stage. Note the complete buccal capsule characteristic of this stage (arrow). [file 13071_2014_609_MOESM1_ESM.tif]

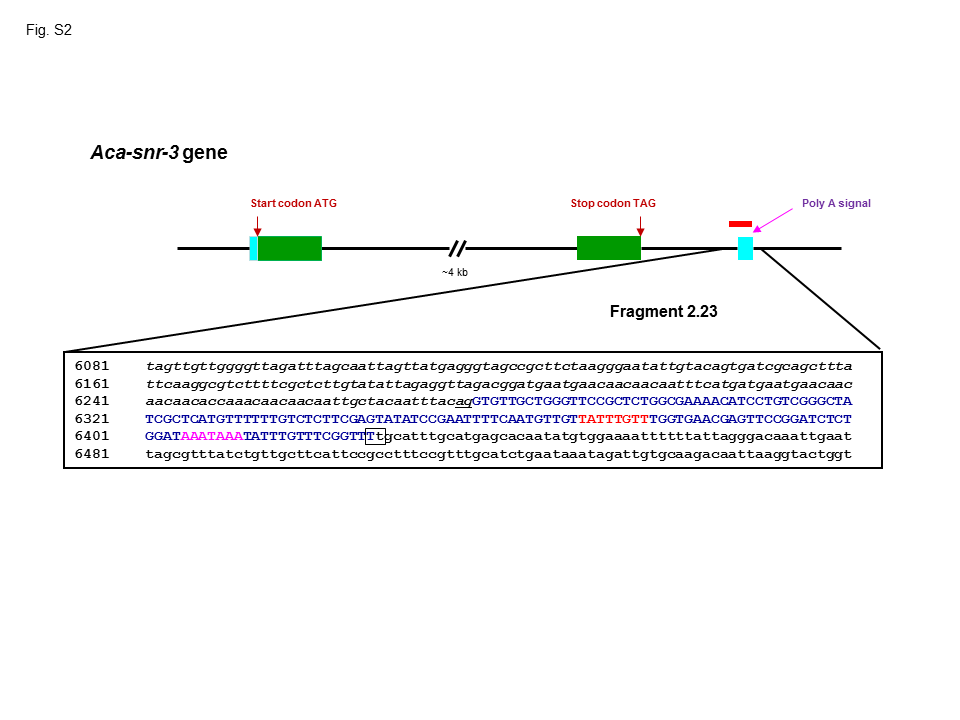

Supplement: Additional file 2: Figure S2. — Map of the Aca-snr-3 gene showing the location of Fragment 2.23 and the Daf-16 binding element. Exons are indicated by boxes, with coding regions in green and untranslated regions in aqua. Introns are indicated as lines. The red bar shows the approximate location of Fragment 2.23, and the inset shows its DNA sequence. The 3′ sequence of intron 2 is in italicized lowercase text, exon 3 sequence is in blue uppercase text, and the DBE in red text. The 3′ splice sequence of intron 2 is underlined, the polyadenylation signal sequence is in pink text, and the site of polyA tail addition is boxed. Numbering is from the original contig sequence. [file 13071_2014_609_MOESM2_ESM.tif]

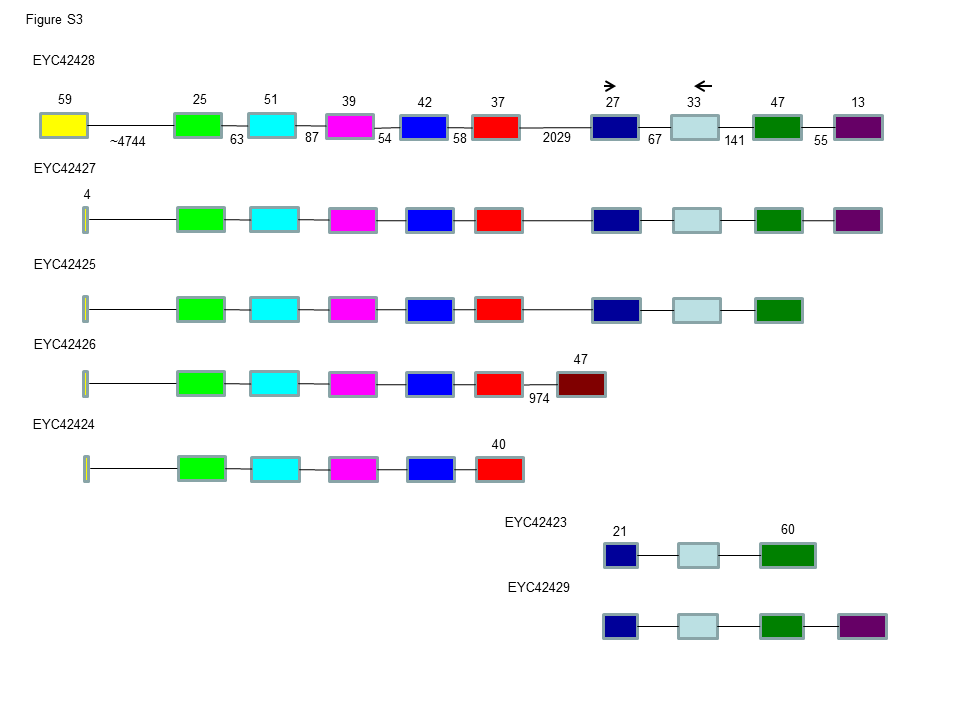

Supplement: Additional file 3: Figure S3. — Predicted isoforms of Ace-LPP-1. Isoforms sequences, indicated by accession number, were obtained from Genbank and mapped to A. ceylanicum draft genomic contigs to determine gene structure. Similar exons are shown in the same color. The number of amino acids is shown above each exon. The intron length in base pairs is indicated below each intron. The location of the primers used for qPCR is indicated by arrowheads. [file 13071_2014_609_MOESM3_ESM.tif]
